# Mechanics Insights of Alpha-Lipoic Acid against Cardiovascular Diseases during COVID-19 Infection

**DOI:** 10.3390/ijms22157979

**Published:** 2021-07-26

**Authors:** Luc Rochette, Steliana Ghibu

**Affiliations:** 1 Equipe d’Accueil (EA 7460), Physiopathologie et Epidémiologie Cérébro-Cardiovasculaires (PEC2), Faculté des Sciences de Santé, Université de Bourgogne-Franche Comté, 21000 Dijon, France; luc.rochette@u-bourgogne.fr; 2 Department of Pharmacology, Physiology and Pathophysiology, Faculty of Pharmacy, “Iuliu Haţieganu” University of Medicine and Pharmacy, 400349 Cluj-Napoca, Romania

**Keywords:** COVID-19, SARS-CoV-2, alpha-lipoic acid, cardiovascular disease, oxidative stress, inflammation

## Abstract

Coronavirus disease 2019 (COVID-19) was first reported in Wuhan, China, in late December 2019. Since then, COVID-19 has spread rapidly worldwide and was declared a global pandemic on 20 March 2020. Cardiovascular complications are rapidly emerging as a major peril in COVID-19 in addition to respiratory disease. The mechanisms underlying the excessive effect of severe acute respiratory syndrome coronavirus 2 (SARS-CoV-2) infection on patients with cardiovascular comorbidities remain only partly understood. SARS-CoV-2 infection is caused by binding of the viral surface spike (S) protein to the human angiotensin-converting enzyme 2 (ACE2), followed by the activation of the S protein by transmembrane protease serine 2 (TMPRSS2). ACE2 is expressed in the lung (mainly in type II alveolar cells), heart, blood vessels, small intestine, etc., and appears to be the predominant portal to the cellular entry of the virus. Based on current information, most people infected with SARS-CoV-2 virus have a good prognosis, while a few patients reach critical condition, especially the elderly and those with chronic underlying diseases. The “cytokine storm” observed in patients with severe COVID-19 contributes to the destruction of the endothelium, leading to “acute respiratory distress syndrome” (ARDS), multiorgan failure, and death. At the origin of the general proinflammatory state may be the SARS-CoV-2-mediated redox status in endothelial cells via the upregulation of ACE/Ang II/AT1 receptors pathway or the increased mitochondrial reactive oxygen species (mtROS) production. Furthermore, this vicious circle between oxidative stress (OS) and inflammation induces endothelial dysfunction, endothelial senescence, high risk of thrombosis and coagulopathy. The microvascular dysfunction and the formation of microthrombi in a way differentiate the SARS-CoV-2 infection from the other respiratory diseases and bring it closer to cardiovascular diseases like myocardial infarction and stroke. Due the role played by OS in the evolution of viral infection and in the development of COVID-19 complications, the use of antioxidants as adjuvant therapy seems appropriate in this new pathology. Alpha-lipoic acid (ALA) could be a promising candidate that, through its wide tissue distribution and versatile antioxidant properties, interferes with several signaling pathways. Thus, ALA improves endothelial function by restoring the endothelial nitric oxide synthase activity and presents an anti-inflammatory effect dependent or independent of its antioxidant properties. By improving mitochondrial function, it can sustain the tissues’ homeostasis in critical situation and by enhancing the reduced glutathione it could indirectly strengthen the immune system. This complex analysis could open a new therapeutic perspective for ALA in COVID-19 infection.

## 1. Introduction

The coronavirus disease 2019 (COVID-19) pandemic represents the most significant public health emergency of the century, and it is caused by the severe acute respiratory syndrome coronavirus 2 (SARS-CoV-2). Novel coronavirus (2019-nCoV)-associated pneumonia cases first appeared in Wuhan, Hubei Province, China, in December 2019. In the following months, SARS-CoV-2 rapidly spread throughout China and the world. Between the index case in early December 2019 and 1 December 2020, >65,000,000 cases and >1,500,000 deaths due to COVID-19 were reported worldwide [1]. 

COVID-19 is a systemic disease that can lead to pneumonia, respiratory failure, and “acute respiratory distress syndrome” (ARDS). ARDS is caused by lung inflammation and increased alveolar endothelial and epithelial permeability, resulting in pulmonary edema with severe hypoxemia. Thus, the lung impairment involves injury in the endothelial and epithelial barriers of the lung [1]. 

Furthermore, SARS-CoV-2 affects the cardiovascular, renal, cerebrovascular, and blood coagulation systems. The clinical cardiovascular manifestations of COVID-19 mainly include cardiac injury: myocarditis, arrhythmia, heart failure, acute myocardial infarction (AMI) and shock. Cardiogenic, septic, or mixed shock is one of the criteria of critical illness in COVID-19. Acute cardiac injury has been associated with cardiac dysfunction and malignant arrhythmias, and those patients with acute cardiac injury exhibited a significantly higher risk of mortality [2]. Thus, the pathology of COVID-19 results in both direct and indirect injuries. The mechanisms underlying the cardiovascular manifestations of COVID-19 are probably multifactorial. Direct injuries are triggered by infection of target cells by the virus. Indirect injuries come from inflammatory reaction, tissue ischemia, hypoxia and from the activation of the immune response and clotting. COVID-19 has been associated with proinflammatory and prothrombotic conditions that can result in thromboembolic events; higher markers of thrombosis have been associated with worse clinical outcomes [3]. Additionally, in some situations patients have developed cardiovascular complications after recovery from COVID-19; the long-term consequences of SARS-CoV-2 infections are not yet fully known [4].

Given our knowledge from prior pandemics, such as the severe acute respiratory syndrome coronavirus (SARS-CoV) in 2002–2004, H1N1 influenza in 2009, the Middle East respiratory syndrome coronavirus (MERS-CoV) in 2012, and Ebola in 2014–2016, as well as scientific advancements, major progress has been made in evaluating multiple strategies for treating SARS-CoV-2 viral infection. Concerning the SARS-CoV-2 therapy, drugs can target various phases of viral life cycle: adhesion and viral entry to host cell, viral protease, inhibition of “cytokine storm” and protection of the various organs. Recently, it has been reported that 1500 clinical trials related to COVID-19 have been registered, but none of the present strategies represents a perfect option. Preventive measures are the best strategy in COVID-19; vaccines and monoclonal antibodies against SARS-CoV-2 have been developed in the meantime [5,6]. 

In addition to the standard clinical trials, new specific therapeutic strategies are being explored that target the reactive oxygen species (ROS) pathway or the restortion of the cellular redox balance. The pathophysiology underlying critical cases of viral pneumonia is associated with severe oxidative stress (OS). It is, therefore, important to find the modalities to decrease the OS pathways activated in COVID-19. OS triggers several biochemical pathways, including nuclear factor kappa B (NF-κB), which is a target for modulating inflammatory responses and regulates multiple phases of immune functions [7]. Antioxidants and inhibitors of NF-κB signaling should be explored as part of a multi-layered approach to COVID-19 treatment. Moreover, in the immune system, antioxidant drugs protect the host cells against OS associated with the infection. 

In this context, a compound such as the alpha-lipoic acid (ALA), which is a potent antioxidant and an inhibitor of NF-κB activation [8,9], may boost human host defense against SARS-CoV-2 and could play a vital role in the treatment of patients.

## 2. Pathogenesis of COVID-19 and Mechanisms of SARS-CoV-2-Induced Organ Diseases

SARS-CoV-2 is a member of the genus *Betacoronavirus.* It is the third coronavirus to human severe respiratory diseases, following the previously identified SARS-CoV and MERS-CoV [10,11,12]. 

Coronaviruses (CoVs), which are a great family of single-stranded enveloped RNA viruses, were not recognized as being very pathogenic in humans until the eruption of SARS caused by SARS-CoV in 2002. Coronaviruses can be divided into four genera: α, β, γ and δ, of which only α and β-coronaviruses are known to infect humans. These viruses have a positive-sense RNA genome that in SARS-CoV-2 encodes 16 nonstructural proteins (NSPs) and 4 structural proteins. The NSPs are essential for replicative functions such as RNA polymerization by the RNA-dependent RNA polymerase. The four structural proteins known as spike (S), envelope (E), membrane (M) and nucleocapsid (N) proteins form the envelope of the virus [13,14]. 

Coronaviruses have a crown-like morphology constituted in particular by S protein (S protein trimers) and which in fact represents the key factor responsible for the entry of the virus into the host cell (the viral infection) [15]. 

N protein is one of the most abundant proteins of CoVs and it presents multiple functions. N-protein surrounds the viral genome, thus protecting it from harsh intracellular or extracellular conditions, but also interferes with replication and propagation of the virus [16]. Additionally, it suppresses type I interferon (IFN-I) signaling in infected human by reducing cellular antiviral defense [17] and upregulates cyclo-oxygenase-2 (COX-2) expression with a high synthesis of proinflammatory mediators [16].

## 3. ACE2-Mediated SARS-CoV-2 Viral Toxicity

**Figure 1:**
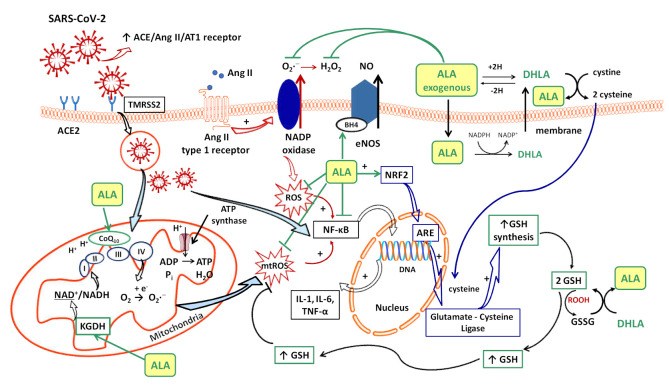
The implications of SARS-CoV-2 infection in cellular redox status and cellular homeostasis, the main therapeutic targets of ALA in this context. The SARS-CoV-2 infection is initiated by the binding of S protein to ACE2 (the receptor of SARC-CoV-2), followed by its priming to the cell membrane (via TMRSS2) and by virus endocytosis. During infection, the ACE-Ang II-AT1 receptor signaling pathway is overactivated, which in turn activates NADPH oxidase with the increase production of O_2_•^−^. The enhancement in mtROS synthesis adds to this, both processes influencing the cellular redox status, endogenous antioxidant level and, subsequently, cellular homeostasis. In addition, the excess of ROS contributes to the activation and nuclear translocation of NF-κB factor, increasing production of pro-inflammatory cytokines and enhancing the pro-inflammatory process. Intracellularly, ALA is reduced to DHLA. The ALA/DHLA redox system interferes with several signaling pathways of OS, even with the inflammatory process, and may present some protection in a SARS-CoV2 infection. Due to its wide distribution, ALA can directly scavenge the ROS, or can restore the reduced form of glutathione or CoQ10, an important constituent of the mitochondrial electron transport chain, thus enhancing endogenous antioxidant protection. Moreover, ALA stimulates GSH synthesis by increasing cellular cysteine uptake and by activating the NRF2-ARE signaling pathways, increasing glutamate–cysteine ligase activity. As cofactor of mitochondrial KGDH, ALA influences the supply with reducing equivalents (NAD^+^/NADH). Dependent on its antioxidant properties, but also independent of them, the ALA/DHLA system inhibits NF-kB signaling with the decrease in pro-inflammatory cytokine secretion. By preventing the oxidative depletion of BH4, an essential cofactor of eNOS, ALA can restore the eNOS activity with an increase of NO bioavailability, and thus, it improves the endothelial function. (ACE2—angiotensin-converting enzyme 2; ADP—adenosine diphosphate; ALA—alpha-lipoic acid; Ang II —angiotensin II; ARE—antioxidant response element, region of the nucleus; AT1 receptor—angiotensin II type 1 receptor; ATP—adenosine triphosphate; BH4—tetrahydrobiopterin; reduced CoQ10—reduced Coenzyme Q10 or ubiquinol; DHLA—dihydrolipoic acid; DNA—deoxyribonucleic acid; e^−^—electron; eNOS—endothelial nitric oxide synthase; GSH—reduced glutathione; GSSG—oxidized glutathione; H—Hydrogen; H^+^—proton; H_2_O—water; H_2_O_2_—hydrogen peroxide; IL-1—Interleukine-1; IL-6—Interleukine-6; KGDH—α-ketoglutarate dehydrogenase; mtROS—mitochondrial ROS; NAD^+^—oxidized nicotinamide adenine dinucleotide; NADH—reduced nicotinamide adenine dinucleotide; NADP^+^—oxidised nicotinamide adenine dinucleotide phosphate; NADPH—reduced nicotinamide adenine dinucleotide phosphate; NF-κB—nuclear factor kappa B; NO—nitric oxide; NRF2—nuclear factor erythroid 2-related factor 2; NRF2-ARE pathway—nuclear factor erythroid 2-related factor 2—antioxidant response element; O_2_—oxygen; O_2_•^−^—anion superoxide; OS—oxidative stress; P_i_—inorganic phosphate; ROS—reactive oxygen species; ROOH—hydroperoxide; SARS-CoV-2—severe acute respiratory syndrome coronavirus 2; TMRSS2—transmembrane proteases like serine protease type 2; TNF-α—tumor necrosis factor-α; I—complex I of the mitochondrial respiratory chain; II—complex II of the mitochondrial respiratory chain; III—complex III of the mitochondrial respiratory chain; IV—complex IV of the mitochondrial respiratory chain; ⊥—inhibition).

SARS-CoV-2 infection is caused by the binding of the viral surface S protein to the human angiotensin-converting enzyme 2 (ACE2). Thus, the S protein has a pivotal role in virus attachment, cell entry and disease pathogenesis, while the ACE2 is considered the specific receptor for SARS-CoV-2. The S protein consists of two subunits: the S1 subunit contains a receptor-binding domain (RBD) that binds to ACE2 on the surface of host cells, while the S2 subunit mediates the fusion between the virus membrane and that of the host cell. The two subunits are released after S protein cleavage by the specific cellular transmembrane proteases like serine protease type 2 (TMPRSS2) or furin [15]. Then follows virus clathrin-mediated endocytosis with intracellular virus integration, its replication, and the initiation of infection in the human host cells (Figure 1) [18]. 

ACE2 is a homolog of angiotensin-converting enzyme (ACE), and it converts angiotensin (Ang) II to angiotensin 1 to 7 (Ang 1-7), thereby diminishing vasoconstriction mediated by the renin angiotensin system (RAS). In the classical RAS cascade, the decapeptide Ang I is converted to Ang II by ACE, which, thus obtained, determines the most specific effects by stimulation of angiotensin II type 1 receptor (AT1 receptor). Therefore, AT1 receptor is one of the key players in the RAS; it promotes various intracellular signaling pathways through NADPH oxidase and ROS [19]. Therefore, the use of angiotensin-converting enzyme inhibitors and angiotensin receptor blockers is common in cardiovascular diseases (hypertension, coronary artery disease, congestive heart failure) and in cardiovascular diseases associated with metabolic disorders like diabetes mellitus. However, there are conflicting data on whether these drugs increase or have minimal effect on ACE2 levels [20].

ACE2, like ACE, is a transmembrane zinc metalloprotease, widely expressed in a variety of mammalian tissues, including the brain [21,22]. ACE2 exerts several protective effects in organs, but genetic ACE2 deficiency is also associated with the development of some diseases. Thus, in ACE2 knockout mice, the development of myocardial hypertrophy and interstitial fibrosis has been noted [23], while in the metabolism field, ACE2 stimulates insulin secretion and attenuates insulin resistance [24]. However, first of all, ACE2 is a well-characterized negative regulator of the RAS, as it converts Ang II into the vasodilatory fragment Ang 1-7, which simultaneously decreases the Ang II concentration to further facilitate the antihypertensive and cardioprotective effects [25]. Ang 1-7 activates the G protein-coupled Mas (mitochondrial assembly) receptor (MasR), inducing many beneficial cardiovascular actions, such as vasodilation, inhibition of cell growth, and antithrombotic effect. The ACE2/Ang 1-7/MasR axis is known to have crucial roles in both the cardiovascular system and the immune system [26]. The dysfunction of the ACE2/Ang 1-7/MasR pathway intensifies inflammation and contributes to the impaired function of the inflamed tissue. 

ACE2 and MasR are highly expressed in the lungs, kidneys, heart, blood vessels and gastrointestinal tract. ACE2 expression is thought to be one of the major factors involved in the biological mechanism underlying tissue-specific infection. ACE2 is expressed primarily in alveolar epithelial type II cells in the normal adult lung. These cells produce surfactant proteins that reduce surface tension, preventing the alveoli from collapsing [27]. The expression of ACE2 in the heart and coronary arteries is even higher than in the lungs. At the single-cell level, ACE2 is highly expressed in pericytes of adult human hearts. Single-cell RNA sequencing data have also revealed that cardiomyocytes (especially those in the right ventricle) express ACE2 at a lower level than pericytes [24,28]. 

TMPRSS2, highly expressed in various organs, acts in conjunction with ACE2. TMPRSS2 activates S protein by its cleavage, both membrane enzymes mediating virus entry into human host cells [29]. Thus, even in the absence of underlying comorbidities, the vital organs are vulnerable to SARS-CoV-2 infection, and they are an important target for this new coronavirus.

On the other hand, along with virus endocytosis, the internalization of ACE2 takes place, which leads to the reduction of cell surface ACE2 enzyme number with the progressive weakening of ACE2-mediated tissue protection and the enhancing of ACE-Ang II-AT1 receptor axis mediated effects. Thus, it seems that ACE2 deficiency plays a central role in ARDS and cardiovascular complications caused by SARS-CoV-2. Additionally, low ACE2 expression associated with age or type II diabetes mellitus may explain the severe lung injuries or severe forms of COVID-19 reported in these situations [30].

## 4. Endothelial Cell Damage: Endothelialitis

It has now been demonstrated that SARS-CoV-2 uses ACE2 for cell entry. Additionally, the proteases TMPRSS2 or furin are critically involved in SARS-CoV-2 cell entry and the virus becoming functional (the infection process) [18,28]. SARS-CoV-2 infection is triggered by binding of the viral S protein to human ACE2, whereas TMPRSS2 induces S protein priming.

Endothelial cells are heavily involved in COVID-19 pathology [4]. Thus, endothelial cells are a direct target of SARS-CoV-2 infection, and they also represent the starting point for the major disorders that accompany this infection [31]. In this field, a pronounced endothelialitis (inflammation within the endothelium due to an immune response) and the local recruitment of inflammatory cells was demonstrated [32]. 

ACE2 counterbalances the vasoconstriction induced by activation of the ACE-Ang II-AT1 receptor axis of the RAS. Overactivation of the ACE-Ang II-AT1 receptor axis of the RAS and the endothelialitis caused by the viral infection will promote vasoconstriction, inflammation, and thrombosis in the vascular bed, where it will cause various tissue injuries, contributing to the mortality of a SARS-CoV-2 infection. A viral and inflammatory endothelialitis may play a key role in the pathogenesis of COVID-19, while the advanced stages of COVID-19 resemble a microvascular disease associated with multiple organ failure [33].

**Figure 2:**
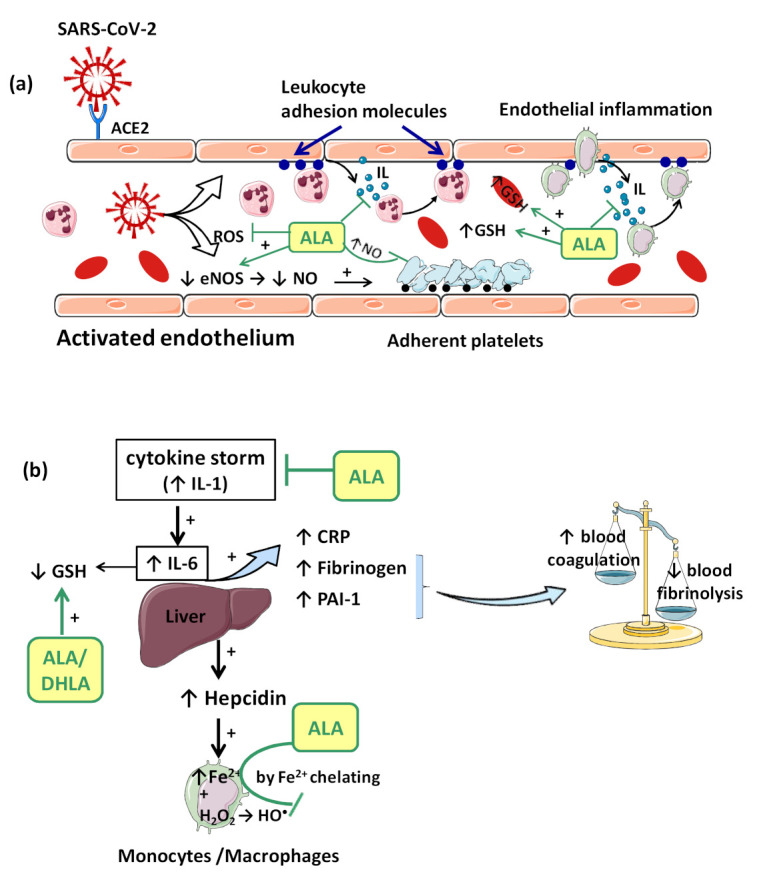
The main vascular implications of SARS-CoV-2 infection and the possible therapeutic targets of ALA. (**a**) Endothelial cells activated by SARS-CoV-2 infection, increase expression of adhesion molecules, pro-inflammatory cytokines and release the VWF. All these promote leukocyte (neutrophils and monocytes) recruitment while amplifying vascular inflammation and supporting platelet adhesion and aggregation with high incidence of arterial thrombosis. (**b**) In addition to a generalized hyper-inflammatory state, the cytokine storm induces hepatic synthesis of fibrinogen, PAI-1 or hepcidin. Hepcidin dysregulates iron homeostasis (decreases iron flow into extracellular fluid), enhancing OS and oxidative injuries. An inverse relationship between IL-6 and GSH levels has also been reported in COVID-19 patients. Due the increase in fibrinogen and PAI-1 levels, an imbalance between coagulation and fibrinolysis pathways is induced with a high risk for venous thrombosis. By scavenging ROS, restoring reduced glutathione, or chelating intracellular Fe^2+^, ALA can contribute to endothelial protection in COVID-19 patients. (ACE-2—angiotensin-converting enzyme 2; ALA—acid alpha-lipoic; CRP—C reactive protein; DHLA—dihydrolipoic acid; eNOS—endothelial nitric oxide synthase; Fe^2+^—ferrous ion; GSH—reduced glutathione; H_2_O_2_—hydrogen peroxide; HO•—hydroxyl radical; IL—interleukins or cytokines; IL-1—Interleukine-1; IL-6—Interleukine-6; NO—nitric oxide; OS—oxidative stress; PAI-1—plasminogen activator inhibitor-1; ROS—reactive oxygen species; VWF—Von Willebrand Factor; ⊥—inhibition).

In all these situations, the activation of endothelial cells takes place with the increased expression of pro-inflammatory cytokines, adhesion molecules and matrix metalloproteinases that amplify the local inflammatory status and disrupt the endothelial barrier function. In addition to capillary leakage with alveolar edema occurrence, these endothelial disturbances sustain platelet adhesion and aggregation, with a high incidence of arterial thrombosis that can predispose to stoke, acute coronary syndrome, or myocardial ischemia/infarction [31,33]. Von Willebrand Factor (VWF), a pro-adherent glycoprotein released from activated endothelial cells due the endothelial injuries, is significantly elevated in plasma COVID-19 patients compared to normal individuals, thus predicting a risk of arterial thrombosis (Figure 2a) [31]. Thus, the endothelialitis contributes to the microcirculatory changes, and indirectly to micro- and macro-thromboses reported in COVID-19 infection (Figure 2a,b). Furthermore, extended into systemic circulation, it could lead to acute myocardial injury. Therefore, endothelialitis plays a key role in the pathogenesis of the pulmonary and cardiovascular complications of COVID-19 [30]. In addition, induction of apoptosis and pyroptosis might have an important role in endothelial cell injury in patients with COVID-19 with the loss of endothelial barrier stability and vascular integrity, emphasizing more alveolar edema and ARDS [34].

## 5. COVID-19 and “Cytokine Storm”

In addition to all disturbances already mentioned, the “cytokine storm” observed in patients with severe COVID-19 contributes to further destruction of the endothelium, leading to ARDS and multiorgan failure. Inflammation and “cytokine storm syndrome”, resembling hemophagocytic lymphohistiocytosis (HLH)/macrophage activation syndrome (MAS), leads to extreme morbidity and mortality due to the uncontrolled activation and proliferation of lymphocytes and macrophages. MAS has been reported in deaths related to autoimmune disorders and infections that are primarily of viral origin [35,36]. 

The term “cytokine storm” was first coined in 1993 to describe graft versus host diseases, and it is characterized by an uncontrolled and unrestricted production of pro-inflammatory cytokines and by a systemic hyper-inflammatory reaction. Cytokines are divided into three subgroups: pro-inflammatory cytokines, including interleukin (IL)-1β (IL-1β), IL-1α, IL-2, IL-17, IL-18, interferon-γ (IFN-γ), and tumor necrosis factor-α (TNF-α); anti-inflammatory cytokines, such as IL-4 and IL-10; and cytokines that have both pro-inflammatory and anti-inflammatory activity, such as IL-6. The cytokines constitute an essential and important part of the body’s immune response, but a “cytokine storm” is nearly always pathogenic, because of its detrimental effects on the host. The term has since been extended to describe the similar sudden cytokine releases associated with autoimmune diseases, sepsis, cancers, acute immunotherapy responses, and infectious diseases [37]. 

Consequences of viral infection of the respiratory epithelium include dysfunction and destruction of alveolar epithelium, and the increase in capillary endothelium permeability. These effects are mediated in part by the impact of the pro-inflammatory “cytokine storm” that is produced during the late antiviral innate immune response. The “cytokine storm” with a generalized hyperinflammatory state appears as a consequence of temporary failure of immune response mediated by type I interferons (IFNs) (e.g., IFN-α, IFN-β, etc.) during the initial period of SARS-CoV-2 infection [37]. In this context, IL-1 seems to represent the trigger factor to which IL-6 later joins [37]. IL-1 can induce its own gene expression, but it also stimulates the synthesis of other pro-inflammatory cytokines (TNF-α, IL-6) and chemokines, thus amplifying the cytokine overproduction and tissue infiltration of leukocytes. Moreover, IL-6 promotes an acute phase response characterized by increased hepatic synthesis of fibrinogen, plasminogen activator inhibitor-1 (PAI-1) and C reactive protein (CRP) (Figure 2b). Thus, besides affecting the integrity and function of endothelium, the “cytokine storm” is associated with a prothrombotic and antifibrinolytic imbalance with a hypercoagulable state responsible for venous thrombosis (blood clot) and for pulmonary embolism risk (Figure 2b) [33,37]. 

It has been reported that markers of systemic inflammation such as CRP, monocyte chemoattractant protein1 (MCP-1) and IL-6 are elevated in patients with poor clinical outcomes and in those with the need for mechanical ventilation, being correlated with the severity of pneumonia and the mortality rate [31,37]. 

Complementarily, pro-inflammatory cytokines such as TNF-α and IFN-γ are highly upregulated in COVID-19 patients, providing another amplification loop of “cytokine storm” and participating in cell death, tissue and organ damages [38,39]. Moreover, TNF-α and IFN-γ induce the increase of nitric oxide (NO) production in endothelial cells via inducible NO synthase (iNOS) activation. This process has been demonstrated in COVID-19 infection. The protection observed in vivo during SARS-CoV-2 infection induced by neutralizing TNF-α and IFN-γ suggests that inhibition of TNF-α and IFN-γ signaling and the reduction of iNOS activity might be beneficial in “cytokine storm” syndromes [40]. 

Among the inductors of iNOS, the lipopolysaccharides (LPS), a component of the outer cell wall of Gram-negative bacteria, has the ability to elicit inflammatory response syndrome. There is a link between high LPS levels in the blood and metabolic syndrome, which predisposes patients to severe COVID-19. An interaction between the S protein and LPS is demonstrated to lead to intensified inflammation [41]. 

In the induction of endothelialitis initiated in COVID-19 patients, pathogenetic factors are involved, including innate immunity factors. The host immune system has multiple innate immune receptors, and innate immunity is a crucial component of preventing virus invasion. The suppressor of the cytokine signaling proteins (SOCS) family is one of the main regulators of the innate immune response. Cytokines activate the innate immune response and initiate a specific immune response against viruses, but dysregulation of host cytokine signaling during disease infection causes organ dysfunction. The SOCS family is a class of negative regulators induced by cytokines, which can block the signal transduction of cytokines [42]. SOCS consist of eight intracellular proteins, SOCS1 to SOCS7, and cytokine-inducible Src homology 2 protein (CIS). SOCS1 and 3 (SOCS1/3) function as virulence factors being used or upregulated by different virus strains or even by bacterial LPS. The consequence of this process is the suppression of type I (IFN-α, IFN-β) and type II (IFN-γ) interferons induction with facilitating the invasion of the virus [37]. Thus, SOCS1/3 antagonist is proposed as a prophylactic and/or therapeutic agent against the COVID-19 diseases, acting by an early interferon release to inhibit the virus replication in the initial stages of infection. This could prevent further stages of viral infection like “cytokine storm” that are much more severe and more difficult to treat. The first use of SOCS1/3 antagonist in a virus infection involved the double-stranded DNA virus, herpes simplex virus type 1 (HSV-1), but it can also inhibit the type A influenza virus [43]. 

## 6. COVID-19, Inflammation and Oxidative Stress

Hypoxemia is an important manifestation of COVID-19, and results in an insufficiency of oxygen supply to organs with a high demand for oxygen and energy, such as the heart. In COVID-19 patients, the imbalance of oxygen supply and demand caused by the inflammatory response and by endothelial dysfunction is similar to the pathophysiology of type 2 myocardial infarction [44]. However, the production of ROS and redox injuries is increased, exacerbating even further the pre-existing disorders, and potentially inducing chronic endothelial dysfunction [45]. 

Several sources responsible for ROS synthesis have been reported in COVID-19; the starting point appears to be the endothelial cells, both by NADPH oxidase (NOx) and the electron leakage from the mitochondrial respiratory chain [4]. The anion superoxide (O_2_•^−^) produced via NOx, due the upregulation of Ang II/AT1 receptor pathway [46] together with the hydrogen peroxide (H_2_O_2_) and peroxynitrite anion (ONOO^−^) subsequently formed, or the direct interactions between viral and mitochondrial proteins, initiates a mitochondrial electron transport chain dysregulation (complex I and III) with a significant increase in mitochondrial ROS production [4]. Furthermore, this excess of ROS triggers the proinflammatory cytokine synthesis (IL-1β, IL-6, IL-18, TNF-α) through activation of NF-κB factor [4] and promotes a proinflammatory endothelial status (Figure 1). 

In consequence, it is reasonable to assume that the endothelium contributes to COVID-19-associated vascular inflammation, particularly endothelialitis, in the various organs, endothelial cells being a key player in this new pathology. Moreover, through the endothelial cells, the inflammatory cascade promotes leukocyte recruitment and amplifies the local OS. Leukocyte transmigration is an important occurrence in the inflammatory response and involves the recruitment of circulating leukocytes, their adhesion to the endothelial cells, and diapedesis toward damaged tissues (Figure 2a). The immune response through increase in leukocyte activity fuels up the vascular ROS synthesis even if these highly reactive structures also play an important role as cell-signaling molecules for innate immunity and for maintaining endothelial homeostasis [4]. The enhanced ROS synthesis also impairs the local antioxidant defense, as highlighted by deacreases in superoxide dismutase (SOD), glutathione peroxidase (GPx), haem oxygenase activities, and reduced glutathione (GSH) levels [33], and exacerbates the general OS.

Accumulation of mononuclear cells (e.g., monocytes/macrophages system) in the small lung vessels is implicated in endothelial injury [34]. Endothelial cell dysfunction includes the impairment of local metabolic environment with a modification in the production of the vasodilator NO and ROS associated with an upregulation of leukocyte adhesion molecules (E-Selectin, P-Selectin) or intercellular adhesion molecule-1 (ICAM-1). Endothelial exocytosis initiates leukocyte and platelet adherence to the capillary wall and leads not only to vascular inflammation, but also to microthrombosis and microvascular obstruction (Figure 2a) [31]. In this vascular area, there is an increased risk of arterial and venous thrombosis highlighted by alterations of specific biomarkers: VWF, fibrinogen, fibrinogen degradation products, dimerized plasma fragment D (D-dimer) and by increased prothrombin time and activated partial thromboplastin time. The biomarkers are implicated in signaling pathways on endothelial cells and circulating cells [47]. High plasma D-dimer levels are associated with a worse prognosis for COVID-19 patients [4]. 

Furthermore, platelet–neutrophil interaction and macrophage activation promote proinflammatory responses including “cytokine storm” and the formation of neutrophil extracellular traps (NETs). High levels of NETs have been reported in hospitalized patients with COVID-19. NETs induce endothelial injury and stimulate both extrinsic and intrinsic coagulation pathways with thrombin activation and clot formation, which amplifies microvascular dysfunctions [48]. 

Thus, both arterial and venous thromboses are common in patients with severe COVID-19 (Figure 2a,b). The incidence of venous thromboembolism events is 20–35% while arterial thromboembolism events represented only 4% [31]. The high incidence of thrombotic events in a way differentiates SARS-CoV-2 infection from the other respiratory diseases and brings it closer to cardiovascular diseases like myocardial infarction and stroke [4]. 

Finally, in COVID-19 patients, hemodynamic changes associated with the systemic inflammatory reaction and pro-thrombotic environment contribute to the initiation and development of cardiovascular complications such as myocarditis and respiratory diseases. Additionally, long-term vascular injury associated with SARS-CoV-2 infection can sustain the myocardial damage characterized by a high plasma troponine level [4]. The early evaluation and continued monitoring of these specific diseases throughout the evolution of COVID-19 are very important. The biomarkers of the inflammatory process and the risk of venous thrombosis represented by various cytokines, fibrinogen and D-dimers may be also used to forecast the outcome of the SARS-CoV-2 infection [47,49]. Understanding the profile and variations of specific biomarkers as a function of different COVID-19 outcomes is the aim of a lot of studies in patients. This way, it is possible to validate the stratified risk approach to the care of COVID-19 patients. As we reported previously, markers of systemic inflammation implicating CRP and IL-6 were elevated in patients with poor outcomes, and the origin of the dysregulated release of cytokines in COVID-19 has been ascribed to various factors. It is assumed that the viral replication during the onset of infection resulted in elevated proinflammatory responses. Moreover, the “cytokine storm” produces an excessive inflammatory and immune response. If inflammation is a vital phenomenon of a healthy immune response, dysregulated inflammation can in turn result in major damage to various organs. Through aging, the efficacy of the innate and adaptive immune response declines. This “immunosenescence” plays a central role in the age-related severity of COVID-19 [50,51]. 

Complex interactions between enzymatic activities, erythropoiesis, iron metabolism, hepcidin (a key regulator of the intracellular iron release), and growth/differentiation factor 15 (GDF15) have been demonstrated during the inflammatory process [52]. Within hours from bacterial and viral infections or other inflammatory stimuli, plasma iron concentrations decrease. This response is referred to as “hypoferremia of inflammation” and has been documented in humans. As we reported previously, the common mechanism of hypoferremia of inflammation is a cytokine-driven increase in hepcidin that downregulates ferroportin, and thereby decreases iron flow into extracellular fluid (Figure 2b). Interestingly, although most bacterial and viral infections rapidly increase hepcidin production in humans and mouse models [53], potential mechanisms of the systemic clinical findings of COVID-19 include the dysregulated iron homeostasis, resulting in OS and inflammatory response. Dysregulation of iron homeostasis with higher iron levels may support the progression of viral infections. Evaluating serum ferritin levels in COVID-19 patients may help to predict the outcome of this pathology. Trends and modifications of iron parameters are reported in many clinical studies, ferritin being a very early and non-specific indicator of inflammation [54]. 

Additionally, H_2_O_2_ formed by different pathways can diffuse across cell membranes, interacting with intracellular iron and inducing the synthesis of hydroxyl radical (HO•), one of the most reactive ROS, enhancing OS and oxidative injuries in COVID-19 patients [4]. The HO• formed can further induce telomeric DNA strand breaks with exacerbation of cellular senescence and the occurrence of all local perturbation derived from it (cellular inflammation, endothelial dysfunction, increased cellular susceptibility to the virus, etc.) [4].

## 7. Therapeutic Perspectives

As we reported, COVID-19-endothelitis could explain the systemic impairment of microcirculatory function in different vascular beds and the clinical sequelae derived from it in patients with COVID-19. This hypothesis provides a rationale for therapies to protect the endothelium, with common anti-inflammatory anti-cytokine drugs [55] or cardiovascular protectors such as ACE inhibitors, ATI receptors antagonists (sartans) and statins [18,30].

Previous reviews have enumerated the potential therapeutic targets and strategies, both conventional and alternative, along with vaccine candidates in clinical trials against COVID-19. However, successful completion of drug development may require several years with no guarantee. Alternatively, already-established drugs can be repurposed to treat the COVID-19 infection. The repurposed drugs that can potentially target the fusion of SARS-CoV-2 into the host cells are elaborated upon here. Some of the previous clinical trials using corticosteroids for the treatment of SARS, MERS, and H1N1 have been used. Corticosteroids are broad-spectrum drugs, known to attenuate inflammation. The anti-inflammatory treatment is as important as the antiviral treatment in COVID-19 management [4,37]. Therapies that target the immune response and decrease the cytokine storm in COVID-19 patients have become an application for new clinical trials [56]. Moreover, antithrombotic treatment, especially anticoagulant agents, is provided to prevent micro- or macro-thromboses and microvascular obstructions. The advance of new drugs or repurposing of commercial compounds has recently been emerging. These targets can be of two different types: they can be either of SARS-CoV-2 origin or can belong to the human endogenous systems involved in anti-COVID-19 defense.

Due the fact that the OS establishes the favorable conditions for the virus’ entry into cells and intracellular virus replication, and is also closely related to the proinflammatory status and endothelial dysfunction, it could be considered an important therapeutic target in the fight against this new coronavirus. Generally, by modulating the endogenous redox status, the immune response could be regulated, too [46]. In this regard, priority should be given to antioxidants whose safety profile has already been proven in experimental models or clinical trials, and alpha-lipoic acid (ALA) could be an appropriate and promising candidate for this new therapeutic approach.

### 7.1. Alpha-Lipoic Acid, Antioxidant and Endothelial Protection Effects

**Figure 3:**
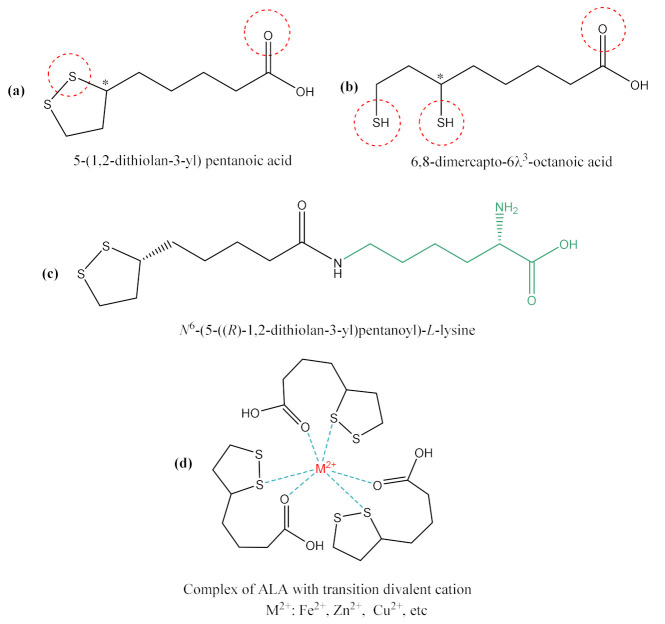
Chemical structures of (**a**) alpha-lipoic acid (ALA), (**b**) dihydrolipoic acid (DHLA), (**c**) lipoyllysine and (**d**) the complex of ALA with transition metals; 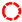
—Potential chelating centers of ALA and DHLA with the transition metal cations; * asymmetric carbon.

ALA or thioctic acid (5-(1,2-dithiolan-3-yl) pentanoic acid) (Figure 3) is a potent and complex antioxidant compound with a very good safety profile [57,58]. Due to the presence of an asymmetric carbon, ALA exists as two enantiomers: the biologically active R-isomer and the S-isomer. Pharmaceutical forms with ALA usually contain a racemic mixture of the R- and S-isoforms, in which S-ALA can enhance the R-ALA’s bioavailability by preventing its polymerization [57]. Moreover, in the last few years, ALA has exceeded the status of antioxidant, its ability to mediate and regulate multiple signaling pathways having been demonstrated. Thus, besides its use as adjuvant therapy for diabetic neuropathy in some European countries, multiple studies have reported the benefits of ALA treatment in metabolic disorders (hyperglycemia, tissue insulin resistance, dyslipidemia or obesity) [59,60,61], endothelial dysfunction [62,63,64,65] or in various inflammatory processes [63,66].

Another great advantage of ALA is represented by its amphiphilic properties, due to which ALA can be distributed both in hydrophilic (plasma, cell cytoplasm, etc.) and in lipophilic (cell membranes, etc.) environments. Actually, the amphiphilic character of ALA is unique among antioxidants [9].

Except for medication, humans obtain the majority of their ALA from food of plant or/and animal origin, and a minor part via de novo mitochondrial synthesis, starting from fatty acids (octanoic acid) and cysteine [9,57]. The available literature data mentions the amounts of ALA contained by certain products of vegetable (spinach, broccoli and tomatoes) or animal (kidney, heart and liver) origin, but, in both situations, there are relatively low amounts of ALA in the dietary sources. In food, R-ALA is covalently bound to lysine from proteins, being found in the form of lipoyllysine (Figure 3) [8,9]. Under physiological conditions, the dietary intake of ALA (R-isomer) joins de novo ALA (R-isomer) synthesis, thus ensuring the proper functioning of basic physiological processes (cofactor of mitochondrial pyruvate dehydrogenase and α-ketoglutarate dehydrogenase (KGDH)) [57]. However, in pathological conditions, especially in complex situations associated with oxidative injuries and a high level of OS, the need for ALA treatment is justified and may be recommended. Depending on the severity of the disease and the state of consciousness of the patient, oral or parenteral (intravenous) pharmaceutical forms of ALA can be used, and the doses can be adjusted, with a daily administration between 600 and 1200 mg ALA frequently being recommended. After oral administration, ALA is rapidly absorbed from the gastrointestinal tract, followed by both its transport into tissues as well as its renal excretion, which explains its very short half-life (30–40 minutes after oral administration and 12 minutes after intravenous administration) [67]. Furthermore, after oral administration, ALA’s bioavailability can be reduced by food intake due to the competition between ALA and nutrients (e.g., medium-chain fatty acids) for the carrier proteins involved in absorption; the H^+^-linked monocarboxylate transporter is the main carrier responsible for the intestinal absorption of ALA, but the Na^+^-dependent multivitamin transporter can also be involved [57]. In concert with the multiple possibilities of transport, and with the multitude of pharmaceutical products used, the gastrointestinal absorption of ALA appears to be quite variable. After oral administration of a racemic mixture of ALA, an absorption of only 20–40% was reported [57], which could limit both its bioavailability and its effectiveness on a much larger scale. In this context, ALA could be recommended either 30 minutes before the meal or 2 hours after the meal [68]. Moreover, even if the currently published results are contradictory, there appears to be a difference in ALA’s bioavailability depending on the patient’s sex and age [69,70]. More specifically, a higher maximum plasma concentration (C_max_) was observed in women compared to men [70], as well as in senior adults (average age of 79 years) compared to young adults (average age of 32 years) [69], after oral administration of ALA. These differences in ALA pharmacokinetics in certain categories of populations do not appear to be relevant either clinically or for therapeutic practice [70]. At the opposite pole, if a higher effectiveness of ALA is pursued, the pharmaceutical forms with parenteral administration (e.g., intravenous (i.v.) administration) can be used. They can also be used in emergencies or critical situations. 

Intracellularly (endothelial cells, erythrocytes, etc.), ALA is reduced to dihydrolipoic acid (DHLA, 6,8-dimercapto-6λ^3^-octoanoic acid, Figure 3), which is subsequently extracellularly released and oxidized to ALA. DHLA presents superior antioxidant properties compared to ALA [71] due to the two free thiol groups within its structure, and also contributes to the recycling and prolongation of ALA effects over a longer period of time (Figure 1) [9,72,73].

ALA is a complex antioxidant molecule that can interfere with several signaling pathways of OS. In addition to its ability to directly scavenge the reactive oxygen species (HO•, HClO, ^1^O_2_), the ALA/DHLA redox couple may indirectly provide antioxidant protection through transition metal chelation, especially of divalent metals (iron and copper), and regeneration of the reduced forms of some endogenous antioxidants (vitamin E, vitamin C and glutathione) (Figure 1, Figure 2a,b) [8,9,57]. Unlike the ions in the extracellular fluids, the intracellular iron is more labile and would be more easily involved in redox reactions [74]. Additionally, metal ions such as iron and copper catalyze the electron transfer from one oxygen species to another [75]. Although Fe^2+^ is involved in various physiological processes, in the presence of H_2_O_2_, a mild oxidizing agent, it has great potential to generate, by Fenton reaction, more aggressive free radicals such as HO•, and thus enhances the OS. ALA cannot neutralize H_2_O_2_, but by chelating the low iron concentration, it can prevent the oxidative injury [76]. In the metal complex formed, Fe^2+^ is surrounded by three molecules of ALA [76], the ratio ALA:Fe^2+^ being 3:1, depending on its potential binding sites (S1 from the disulfide bridge, favored in electrophilic reaction due a higher electron density, and the carboxylate group [77]. Both ALA and DHLA have metal-chelating capacity, ALA reacts with Fe^2+^ while DHLA chelates even Fe^3+^ [75,76]. Moreover, by chelating Cu^2+^, the ALA/DHLA system prevents copper-induced lipid peroxidation or Cu^2+^-catalyzed ascorbic acid oxidation with catalase inactivation and increased production of H_2_O_2_ within the erythrocytes [78]. However, the efficacy of ALA depends on the amount of vitamin C and its ability to complex iron or copper or to reduce Fe^3+^ to Fe^2+^ [75,78,79]. Therefore, at high concentrations of vitamin C, the effectiveness of ALA is less obvious or is even reduced, and a dose increase could be required, if possible [75].

Due to these properties, the ALA/DHLA system could attenuate the erythrocyte oxidative injuries and cell lysis derived from these, especially in a context of increased plasma OS, as might be found in COVID-19. Nevertheless, to obtain maximum effectiveness in all aspects of the COVID-19 treatment, the immunity stimulation adjuvant therapy that is frequently used, and which may include high doses of vitamin C and zinc, should be taken into account [79,80]. Thus, in order to avoid drug interactions such as gastrointestinal or plasmatic Zn^2+^ chelation by ALA, it is mandatory to space out the administration of the two pharmaceutical products, possibly with the initial administration of ALA, especially since ALA presents the advantage of a single daily administration and a short half-life.

Furthermore, due to its high redox potential (−320 mV), the ALA/DHLA system provides more protection from oxidative damage, being more effective than the endogenous reduced/oxidized glutathione (GSH/GSSG) system (−240 mV), a basic constituent of the first line of antioxidant defense [8,9]. 

From the vast literature data, the therapeutic potential of ALA in diabetic neuropathy together with its off-label recommendations is summarized in Table 1.

ALA has been safely used in a dose range of 100–1800 mg/day in a multitude of trials (Table 1). However, as it has been used widely, and the dose is increased depending on the pathology, several side effects have been reported. Along with more common side effects like nausea, vomiting, dizziness, and cutaneous rash [58,102], other more severe side effects with clinical significance like hypoglycemia (especially after parenteral administration) or even liver necrosis have been noted [58,103,104]. Therefore, the therapeutic use of ALA would require close and long-term pharmacotoxicological monitoring.

The vascular endothelium seems to be a privileged target for the protective action of ALA. By reducing OS, ALA can restore the endothelial nitric oxide synthase (eNOS) activity with the consequent increase of NO bioavailability and improvement in endothelial function [105]. This is partially done by preventing the oxidative depletion of tetrahydrobiopterin (BH4), an essential cofactor of eNOS (Figure 1) [8,106], and partially by increasing the activity of dimethylarginine dimethylaminohydrolase (DDAH), the metabolizing enzyme of plasma asymmetric dimethylarginine (ADMA) [107]. In this way, on the one hand, eNOS is recoupled, and its function is switched to resuming NO production instead of superoxide anion production [106], while, on the other hand, eNOS inhibition by the endogenous ADMA is canceled [107]. By enhancing NO and decreasing ONOO^−^ levels, the nitric-oxide-dependent vascular relaxation is preserved, and platelet aggregation is limited, too (Figure 2a) [8,108]. Thus, it has been reported that the flow-mediated dilation (FMD) level, an accurate, non-invasive index of endothelial function, was significantly improved under ALA treatment [63,65,105], with the greatest effect being obtained in diabetic patients [62,105].

### 7.2. Alpha-Lipoic Acid Effects in COVID-19

ALA is an antioxidant agent with great prospects for use as adjuvant therapy in COVID-19 patients. More precisely, besides reducing OS and protecting the vascular endothelium, it can diminish the cellular entry of SARS-CoV-2 and the inflammatory process, and it can indirectly stimulate the immune system.

Some studies have mentioned that the disulfide–thiol balance would play an important role in cell–virus interaction processes and the virus’ entry into the cells. OS significantly influences this ratio and the viral infection rate [46]. Thus, ROS can easily oxidize the cysteine residues from S protein (RBD) and membrane ACE2 (peptidase domain), leading to enhanced numbers of disulfide groups and the increase in affinity between the two structures, even with a possible increase in COVID-19 severity in certain situations [46,109]. As we reported previously, the relationship between the ALA/DHLA system and thiol redox status has already been established. On the one hand, it increases the GSH/GSSG ratio significantly, and, by modulation of the intracellular HS-/-S-S- ratio, it activates the insulin-signaling pathway with the increase of intracellular glucose uptake and reduces glycemia [9]. Thus, it is possible that through a similar mechanism, the cell entry of SARS-CoV-2 could be affected in the presence of ALA treatment.

Unfortunately, there are very few data on the effects of ALA as adjuvant therapy in patients with COVID-19; most publications related to ALA and different aspects of COVID-19 are reviews. However, in a small recent study, Zhong et al. evaluated the clinical efficacy and safety of ALA in patients with critical forms of COVID-19. Although the study was performed on a small number of patients and the intravenous treatment with ALA (once daily dose of 1200 mg) was administered only for 7 days, in addition to the standard medical care, the results obtained show a lower all-cause mortality rate in the ALA COVID-19 group than in the placebo group, at 30-day follow-up [110]. Additionally, in a previous study, it was demonstrated in vitro that the enhanced susceptibility to viral infection with Human Coronavirus (HCoV) 229E in glucose-6-phosphate dehydrogenase (G6PD)-deficient cells was ameliorated by ALA; this strengthens even further the hypothesis that a high cellular OS provides a favorable environment for viral replication and virulence, and highlights the role of antioxidants, especially of GSH, in innate immunity [111]. Further studies with a larger patient cohort are needed to highlight and validate the role of ALA in patients with different forms of COVID-19.

Another important effect of ALA, both dependent on its antioxidant properties, as well as independent of them, is the anti-inflammatory effect. By reducing the ROS, the general inflammatory process is also decreased. Moreover, by chelating copper, the ALA/DHLA system inhibits the activation and nuclear translocation of NF-κB, with the decrease in pro-inflammatory cytokine secretion, regardless of its antioxidant properties (Figure 1) [9,57,112]. Due to the low cytokine levels and by inducing heme oxygenase-1 upregulation, ALA subsequently decreases the adhesion molecules’ expression (E-selectine, vascular cell adhesion molecule-1 (VCAM-1), intracellular adhesion molecule-1 (ICAM-1)) and the extravascular leucocyte migration (Figure 2a) [98,113]. A significant number of recent clinical trials [63,66,114] and experimental studies [60,61] have reported the efficacy of ALA in different inflammatory process by decreasing the inflammatory markers such us CRP, IL-6 or TNF-α [115]. In this context, it was noted that ALA contributes to multiple organ protection in sepsis [115], in part through activation of autophagy, too [116].

A relevant contribution of mitochondrial ROS synthesis and mitochondrial dysfunction in COVID-19 pathogenesis has been noted. Thus, some studies have proposed mitochondria to be among the main adjuvant therapeutic targets in sepsis [117] and other acute disorders, including in COVID-19 management [115,118]. ALA is a cofactor of pyruvat dehydrogenase and α-ketoglutarate dehydrogenase (KGDH, the E2 sub-unit), key regulatory enzymes within the Krebs cycle, which ultimately influences the adenosine triphosphate (ATP) production [73,119]. KGDH is a key sensor of mitochondrial redox status, which in turn is inactivated by an elevated level of ROS; it is also the key rate-limiting enzyme for nicotinamide adenine dinucleotide (NADH) production, thus controlling the supply with reducing equivalents or mitochondrial electron transports (Figure 1) [119,120]. A decline in KGDH activity has been reported in a variety of neurological and cardiovascular disorders associated with OS. Moreover, a specific inhibition of KGDH through oxidative reactive products can induce mitochondrial cytochrome c release and cell death [119,120]. Thus, both through its complex antioxidant properties and as a cofactor of KGDH [9,73], ALA could mitigate mitochondrial oxidative damages and improve mitochondrial function with the maintaining of tissues’ homeostasis in all of the above-mentioned pathologies [73,115,117]. Moreover, the ALA/DHLA system is able to reduce ubiquinone (CoQ10) to ubiquinol, an important constituent of the mitochondrial electron transport chain, and to enhance the NAD(P)H/NAD(P)^+^ ratio, a component of cell redox state (Figure 1) [73,108]. 

Glutathione, which is implicated in redox state, is an essential endogenous antioxidant responsible for reducing hydroperoxides and H_2_O_2_ and for preventing cellular oxidative damage. A decrease in circulating or intracellular GSH or GSH/GSSG ratio has been reported in different bacterial or viral infections, in lung function impairment, and in cardiometabolic disorders [121]. Generally, GSH depletion is a direct consequence of viral infection; this aspect is required for virus replication [121]. More precisely, the loss of GSH affects Na^+^ H^+^ membrane antiport with the decrease in intracellular pH, which facilitates both virus endocytosis and its replication. Secondarily, the viruses possess a variety of adaptive mechanisms (activation of NADPH oxidase and NF-κB or down regulation of NRF2 expression), which further reduce the host cell GSH. The GSH deficiency in SARS-CoV2 infection is firstly due to a deficient nuclear translocation of NRF2 and an elevated level of IL-6. An inverse relationship between IL-6 and GHS levels has been reported in COVID-19 patients [121]. Subsequently, a low level of GSH can be responsible for immune dysfunctions (critical activity of natural killer lymphocytes), an increased of viral load and a high susceptibility to viral infection [46]. At the opposite pole, a high level of glutathione is associated with leukocyte proliferation, strengthened immune system, and an effective antiviral protection. By maintaining an endogen high GSH/GSSG ratio, virus replication and severe forms of COVID-19 can be prevented [46]. Thus, an antioxidant agent that keeps or enhances reduced glutathione levels could prevent lymphocyte exhaustion or immune cell depletion due to a chronic hyperinflammation state or to a cytokine excess [121]. Although encouraging results have been obtained with N-acetyl cysteine (NAC) or exogenous GSH, ALA could successfully complete the list of molecules with an important effect on GSH in COVID-19 infection. However, it should taken into account that NAC presents a low cerebral bioavailability, and intracellular delivery of exogenous GSH may be deficient in some tissues (e.g., heart, brain, etc.) due to the lack of specific carriers [108]. In addition to a wide tissue distribution, ALA is a versatile antioxidant that can enhance GSH/GSSG ratio by several mechanisms. The ALA/DHLA system stimulates GSH synthesis by increasing cellular cysteine uptake, a rate-limiting substrate for GSH synthesis, and by activation of the NRF2-ARE signaling pathways with the increased activity of glutamate–cysteine ligase (the rate-limiting enzyme in GSH synthesis). Additionally, its ability to restore GSH from oxidized glutathione should not be neglected (Figure 1, Figure 2a,b) [9,73,108]. GSH levels decrease in relation to age and, additionally, age together with some comorbidities such as diabetes, obesity and hypertension have a major impact on the development of severe forms of COVID-19 [46,121].

Another important aspect of the ALA-GSH relationship that could be also capitalized upon in the context of SARS-CoV-2 infection is the ALA hepatoprotection in acetaminophen-induced hepatotoxicity. Acetaminophen (paracetamol) is one of the most widely used and well-tolerated analgesic antipyretic drugs, but at overdoses, it can induce hepatic necrosis via its highly reactive intermediate metabolite, N-acetyl-para-benzoquinone imine (NAPQI). More specifically, in acetaminophen overdose or when glutathione is depleted by 70%, the excessive amount of NAPQI covalently binds to the hepatic intracellular protein sulfhydryl groups, including those from cytoplasmic or mitochondrial enzymes, and induces hepatic OS, cellular toxicity, and hepatocytolysis, which lead to irreversible hepatic necrosis [122,123]. In these situations, the administration of NAC is authorized, but there are also some experimental studies that highlight the hepatic benefits of ALA both by reducing local OS and by restoring the optimal level of GSH [124,125]. Thus, in patients with COVID-19 and a high dose acetaminophen-treated fever, which do not tolerate NAC or who are not recommended to take mucolytics (expectorants), ALA could be an alternative of NAC. Additionally, due to multiple ways to increase the GSH levels, but also due to multiple processes leading to GSH depletion, the hepatoprotective effect of ALA might be obvious or relevant after a period of successive administrations. Furthermore, administration of ALA during the same period of time as acetaminophen may provide maximum hepatic protection.

## 8. Conclusions

Besides respiratory symptoms, COVID-19 is also considered an endothelial and coagulopathy disorder with severe cardiovascular complications. Furthermore, the main connection between them seems to be the inflammation via mitochondrial and endothelial ROS production. In this context, the structures involved in ROS synthesis and the multiple signaling pathways of OS may be a justified therapeutic target in COVID-19 management. Through the various possibilities of modulating the cellular redox state, ALA could be a potential adjuvant therapy in COVID-19 patients. More randomized control trials are needed to evaluate and confirm the efficacy of ALA in COVID-19 infection. 

## Figures and Tables

**Table 1:** A brief selection of the main effects or benefits of alpha-lipoic acid in various pathologies.

Category of Patients Treated with ALA	Protocols (ALA doses)	Period of Treatment	Effects/Benefits	References
**Diabetic neuropathy**				
Diabetic patients with polyneuropathy	600 mg/day (orally)	4 years	Improvement and prevention of progression of Neuropathy Impairment Score of the lower limbs (NIS-LL).	[81]
Diabetic patients with neuropathy	600 mg/day (orally)	40 days	Reduction of neuropathic symptoms.	[82]
Diabetic patients with polyneuropathy	600 mg/day (i.v.) for 5 days, over 21 days	3 weeks	Improvement of the Total Symptom Score (TSS) of neuropathy.	[83]
**Nephropathy**				
Diabetic patients with nephropathy	600 mg/day (i.v.)	8 weeks	Reducing the serum level of creatinine and urinary albumin excretion. Improvement of endothelium-dependent flow-mediated dilation (FMD). Reducing the plasma OS by decreasing the serum level of malondialdehyde (MDA) and increasing of the superoxide dismutase (SOD) activity.	[84]
**Diabetic patients**				
Diabetic patients	600 mg/day (i.v.)	3 weeks	Decreasing of plasma level of asymmetric dimethylarginine (ADMA).	[85]
Diabetic patients	400 mg/day (orally)	4 weeks	Decreasing the levels of the OS markers.	[86]
Diabetic patients	600 mg (i.v.)	3 weeks	Improving of endothelial-dependent vasodilation.	[65]
**Obesity**				
Obese patients	300 mg/day (orally)	10 weeks	Reduction of the body weight.	[87]
Obese patients	1200 mg/day (orally)	8 weeks	Reduction of the body weight and the waist circumference.	[88]
Obese patients	300 mg/day (orally)	10 weeks	Reducing of the body mass index, fat mass and HOMA-IR (Homeostasis Model Assessment of Insulin Resistance).	[89]
Obese patients	300 mg/day (orally)	10 weeks	Reducing of circulating levels of saturated fatty acids.	[90]
Obese patients with diabetes, hypertension, or hypercholesterolemia	1200 mg/day or 1800 mg/day (orally)	20 weeks	Reduction of the body weight in patients treated with 1800 mg/day ALA.	[91]
**Schizophrenia**				
Patients with schizophrenia	600–1800 mg/day (orally)	12 weeks	Reducing of body mass index and visceral fat mass.	[92]
Patients with schizophrenia	100 mg/day (orally)	4 months	Reduction of Brief Psychiatric Rating Scale (BPRS) scores and extrapyramidal symptoms.	[93]
Patients with schizophrenia	500 mg/day (orally)	3 months	Decreasing of fasting glucose levels and increasing of plasma adiponectin levels.	[94]
**Simultaneous kidney–pancreas transplantation**				
During simultaneous kidney–pancreas transplantation	600 mg (i.v.)	A single dose administered immediately before the surgical procedure	Reducing in plasma inflammatory markers. Decreasing the incidence of the early kidney dysfunction and clinical graft pancreatitis in post-transplant patients.	[95]
**Liver transplantation**				
During liver transplantation	600 mg (i.v.)	A single dose administered just before graft reperfusion	Less inflammatory grafts. Protection against hypoxia and OS. Reducing of post-reperfusion syndrome.	[96]
**Multiple sclerosis**				
Patients with -multiple sclerosis	1200 mg/day (orally)	2 years	Improvement of patients’ walking performances.	[97]
Patients with -multiple sclerosis	1200 mg/day (orally)	12 weeks	Decreasing of plasma pro-inflammatory cytokine levels (ICAM-1, IL-4, INF-γ, TGF-β, Transforming growth factor beta).	[98]
**Chronic subclinical inflammatory state**				
Diabetes patients with a history of non-Q-myocardial infarction	600 mg/day (orally)	4 months	Reducing of systemic inflammation by decreasing of some inflammatory marker levels (C-Reactive Protein, IL-6 and TNF-α).	[99]
Obese patients with atrial fibrillation	600 mg/day (orally)	12 months	Decreasing of plasma inflammatory markers (C-Reactive Protein, TNF-α) and nitrotyrosine levels.	[100]
Obese patients with cardiomyopathy	600 mg/day (orally)	12 months	Decreasing of plasma inflammatory markers (C-Reactive Protein, TNF-α) and OS markers (nitrotyrosine) levels.	[101]

## Abbreviations

2019-nCoV	novel coronavirus 2019
ACE	angiotensin-converting enzyme
ACE2	angiotensin-converting enzyme 2
ADMA	asymmetric dimethylarginine
ADP	adenosine diphosphate
ALA	alpha-lipoic acid
AMI	acute myocardial infarction
Ang	angiotensin
Ang 1-7	angiotensin 1 to 7
ARDS	acute respiratory distress syndrome
ARE	antioxidant response element
AT1	angiotensin II type 1 receptor
ATP	adenosine triphosphate
BH4	tetrahydrobiopterin
BPRS	Brief Psychiatric Rating Scale
CIS	cytokine-inducible Src homology 2 protein
Cmax	maximum plasma concentration
CoQ10	coenzyme Q10 or ubiquinol
COVID-19	coronavirus disease 2019
CoVs	coronaviruses
COX-2	cyclo-oxygenase-2
CRP	C reactive protein
DDAH	dimethylarginine dimethylaminohydrolase
D-dimer	dimerized plasma fragment D
DHLA	acid dihydrolipoic
DNA	deoxyribonucleic acid
E	envelope protein
e^−^	electron
eNOS	endothelial nitric oxide synthase
Fe^2+^	ferrous ion
FMD	flow-mediated dilation
G6PD	glucose-6-phosphate dehydrogenase
GDF15	growth/differentiation factor 15
GPx	glutathione peroxidise
GSH	reduced glutathione
GSSG	oxidized glutathione
H	hydrogen
H^+^	proton
H_2_O	water
H_2_O_2_	hydrogen peroxide
HCoV	human coronavirus
HLH	hemophagocytic lymphohistiocytosis
HO•	hydroxyl radical
HOMA-IR	Homeostasis Model Assessment of Insulin Resistance
HSV-1	herpes simplex virus type 1
I	complex I of the mitochondrial respiratory chain
i.v.	intravenous administration
ICAM-1	intercellular adhesion molecule-1
IFN-I	type I interferon
IFNs	interferons
IFN-γ	interferon-γ
II	complex II of the mitochondrial respiratory chain
III	complex III of the mitochondrial respiratory chain
IL	interleukin
IL-1	interleukine-1
IL-1β	interleukin-1β
IL-6	interleukine-6
iNOS	inducible nitric oxide synthase
IV	complex IV of the mitochondrial respiratory chain
KGDH	α-ketoglutarate dehydrogenase
LPS	lipopolysaccharides
M	membrane protein
MAS	macrophage activation syndrome
MasR	mitochondrial assembly receptor
MCP-1	monocyte chemoattractant protein-1
MDA	malondialdehyde
MERS-CoV	Middle East respiratory syndrome coronavirus
mtROS	mitochondrial ROS
N	nucleocapsid protein
NAC	N-acetyl cysteine
NAD(P)	nicotinamide adenine dinucleotide (phosphate)
NAD(P)H	reduced form of nicotinamide adenine dinucleotide (phosphate)
NAPQI	N-acetyl-para-benzoquinone imine
NETs	neutrophil extracellular traps
NF-κB	nuclear factor kappa B
NIS-LL	Neuropathy Impairment Score of the lower limbs
NO	nitric oxide
NOx	NADPH oxidase
NRF2	nuclear factor erythroid 2-related factor 2
NRF2-ARE	nuclear factor erythroid 2-related factor 2–antioxidant response element
NSPs	nonstructural proteins
O_2_	oxygen
O_2_•^−^	anion superoxide
ONOO^−^	peroxynitrite anion
OS	oxidative stress
PAI-1	plasminogen activator inhibitor-1
P_i_	inorganic phosphate
RAS	renin angiotensin system
RBD	receptor-binding domain
RNA	ribonucleic acid
ROOH	hydroperoxide
ROS	reactive oxygen species
S	spike protein
SARS-CoV	respiratory syndrome coronavirus
SARS-CoV-2	severe acute respiratory syndrome coronavirus 2
SOCS	suppressor of the cytokine signaling proteins
SOD	superoxide dismutase
TMPRSS2	transmembrane protease serine 2
TNF-α	tumor necrosis factor-α
TSS	Total Symptom Score
VCAM-1	vascular cell adhesion molecule-1
VWF	Von Willebrand Factor
